# Driver-Automated Vehicle Interaction in Mixed Traffic: Types of Interaction and Drivers’ Driving Styles

**DOI:** 10.1177/00187208221088358

**Published:** 2022-04-25

**Authors:** Zheng Ma, Yiqi Zhang

**Affiliations:** 183843 Penn State College of Engineering, State College, PA, USA; 311285 Pennsylvania State University, University Park, PA, USA

**Keywords:** automated vehicles, human-automation interaction, aggressive and risky driving, driving style, mixed traffic, driver behavior

## Abstract

**Objective:**

This study investigated drivers’ subjective feelings and decision making in mixed traffic by quantifying driver’s driving style and type of interaction.

**Background:**

Human-driven vehicles (HVs) will share the road with automated vehicles (AVs) in mixed traffic. Previous studies focused on simulating the impacts of AVs on traffic flow, investigating car-following situations, and using simulation analysis lacking experimental tests of human drivers.

**Method:**

Thirty-six drivers were classified into three driver groups (aggressive, moderate, and defensive drivers) and experienced HV-AV interaction and HV-HV interaction in a supervised web-based experiment. Drivers’ subjective feelings and decision making were collected via questionnaires.

**Results:**

Results revealed that aggressive and moderate drivers felt significantly more anxious, less comfortable, and were more likely to behave aggressively in HV-AV interaction than in HV-HV interaction. Aggressive drivers were also more likely to take advantage of AVs on the road. In contrast, no such differences were found for defensive drivers indicating they were not significantly influenced by the type of vehicles with which they were interacting.

**Conclusion:**

Driving style and type of interaction significantly influenced drivers’ subjective feelings and decision making in mixed traffic. This study brought insights into how human drivers perceive and interact with AVs and HVs on the road and how human drivers take advantage of AVs.

**Application:**

This study provided a foundation for developing guidelines for mixed transportation systems to improve driver safety and user experience.

**Précis:** The present study conducted a web-based, supervised experiment. Thirty-six drivers were classified into three driver groups (aggressive/moderate/defensive) and experienced two-block driving tasks in both HV-AV interaction and HV-HV interaction in a balanced sequence. The results revealed that driving style and type of interaction influenced drivers’ subjective feelings and decision making.

## Introduction

Automated vehicles (AVs) represent a rapidly developing technology intended to improve traffic safety (Favarò et al., 2018). As AVs come to market, there will be a transition stage during which AVs and human-driven vehicles (HVs) share the road and drivers’ familiarity with AVs will be based largely on their interactions with AVs when the drivers themselves are driving HVs (Bansal & Kockelman, 2017). Simulation studies have found that the introduction of AVs influences traffic flow and driving safety in mixed AV-HV traffic (Arvin et al., 2020; Papadoulis et al., 2019; Rahman & Abdel-Aty, 2018; Sinha et al., 2020; Virdi et al., 2019; Ye & Yamamoto, 2019; Zheng et al., 2020). However, several survey studies found drivers of HVs may change their driving behavior negatively when they interact with AVs on the road (Chityala et al., 2020; Lee et al., 2021; Liu et al., 2020; Trende et al., 2019). To ensure driver safety, it is necessary to understand how and why drivers change their behavior in mixed traffic.

Many researchers have used the three-level hierarchy of strategic, tactical, and control tasks to model drivers’ driving tasks in manual driving (Michon, 1985; Van Der Molen & Boetticher, 1987). The strategic level concerns general trip planning (Ranney, 1994). The tactical level involves driving negotiation in common scenarios by developing strategies, such as gap acceptance at intersections, while the control level is related to immediate vehicle control inputs, such as acceleration and deceleration. To date, behavioral studies of the impact of AVs on HV driver performance in mixed traffic have focused mainly on drivers’ control-level performance in simple car-following scenarios. This control-level performance does not require complex decision making from human drivers in HVs. Researchers have found that HV drivers drive more smoothly and have extended time-to-collision (TTC) when following AVs than HVs, indicating that AVs contribute to fewer traffic accidents and more stable traffic flow (Mahdinia et al., 2021; Rahmati et al., 2019). By contrast, traffic accident reports of AVs from 2015 to 2020 released by the California Department of Motor Vehicles (DMV) suggest that HVs colliding with the AVs that they are following is a severe problem. For instance, Favarò et al. (2017) analyzed accident reports from the California DMV and found that rear-end collisions at intersections constitute the most frequent type of accidents arising from HV-AV interactions. Petrović et al. (2020) conducted a comparative analysis of traffic crashes arising from HV-AV interactions and HV-HV interactions at multiple locations and found the percentage of rear-end collisions during HV-AV interactions (64.2%) was significantly higher than that of collisions during HV-HV interactions (28.3%). The discrepancy between findings from behavioral studies and accident analyses could be attributed to different driving scenarios and different levels of driving tasks being investigated. Previous behavioral studies focused mainly on simple car-following scenarios that only required drivers’ control-level performance, whereas accident analyses focused mainly on more complex car-following scenarios (e.g., intersections) that required complex decision making from human drivers. Therefore, it is important to conduct behavioral studies to understand, in complex driving scenarios that required drivers’ decision making at the tactical level, how the HV-AV interactions differently influence drivers’ decision making compared to HV-HV interactions.

A few empirical studies have explored changes in HV drivers’ decision making at the tactical level of Ranney’s model in HV-AV interactions. Lee et al. (2021) and Liu and Du (2021) conducted survey studies on drivers’ decision making by presenting a list of hypothetical scenarios. They found that drivers were more likely to behave aggressively in HV-AV interactions compared to HV-HV interactions. Millard-Ball (2018) modeled the interaction between AVs and pedestrians based on game theory and found that pedestrians take advantage of AVs due to AVs’ defensive driving style. Trende et al. (2019) informed drivers about AVs’ defensive driving style and explored the HV drivers’ merging decisions in mixed traffic via a driving simulator. The results showed that under time pressure, drivers merged with a smaller gap and behaved more aggressively in HV-AV interactions than in HV-HV interactions. All these studies show that humans’ decision making is affected by the type of vehicle with which they are interacting. However, little research has investigated how drivers’ driving style influences their decision making when interacting with AVs in mixed traffic.

*Driving style* is defined as a person’s preferred way of driving; over time, a person’s driving style develops into their driving habits (Elander et al., 1993; Kleisen, 2011). Previous research has investigated AV drivers’ driving styles and found that drivers’ driving styles significantly influence their subjective feelings, decision making, and takeover performance (Ma & Zhang, 2021). As AVs are mostly programmed to drive defensively, it is unclear whether HV drivers’ driving styles influence their subjective feelings, decision making, and driving performance when interacting with AVs on the road compared to HVs. A survey study that investigated public attitudes toward AVs found aggressive drivers are more open to AVs than other groups of drivers and the researchers speculate that it is because aggressive drivers think they can take advantage of AVs more easily than human drivers (Tennant et al., 2016). However, their study did not investigate whether aggressive drivers are more likely than other drivers to make aggressive decisions and take advantage of AVs. These studies suggest driving style may be a crucial factor influencing drivers’ decision making in HV-AV interactions; however, further research is necessary.

Drivers’ subjective feelings constitute another important indicator that is associated with driving behavior in mixed traffic (Lee et al., 2021; Ma & Zhang, 2021; Siebert et al., 2013). Several experimental studies have investigated drivers’ subjective feelings when riding with AVs, including anxiety, alertness, comfort, and safety (Bellem et al., 2016, 2018; Hartwich et al., 2018; Koo et al., 2016; Ma & Zhang, 2020, 2021). Ma and Zhang (2021) found that when AVs’ driving styles align with drivers’ driving styles, drivers experience more comfort and perceived safety and engage in fewer takeovers. Koo et al. (2016) conducted a simulator study and found that engaging autonomous actions from partial AVs increased AV drivers’ anxiety and alertness and made them drive more slowly. Although these studies focus on driver-AV interaction within the vehicles rather than HV-AV interactions in mixed traffic, they indicate that subjective feelings may influence HV drivers’ decision making and driving behavior. A survey study done by Lee et al. (2021) is one of the few studies that investigates drivers’ subjective feelings in HV-AV interaction. They found that drivers who rated their subjective feelings (e.g., anger, irritation) negatively were more likely to make aggressive decisions in HV-AV interaction than drivers who did not rate their feelings negatively. However, their study did not investigate the effect of driving style on drivers’ subjective feelings in HV-AV interaction.

The present study focuses on mixed traffic in which HVs share the road with Level 5 fully AVs. It aims to investigate the impact of drivers’ driving style (i.e., aggressive, moderate, or defensive) and type of interaction (HV-AV vs. HV-HV) on drivers’ decision making and subjective feelings. Compared with driver decision making in HV-HV interaction, driver decision making in HV-AV interaction was investigated in two blocks: drivers’ intention to behave aggressively in car-following scenarios when they first interact with AVs and drivers’ tendency to take advantage of AVs in other, more complex scenarios after becoming familiar with the AVs’ behavior. It is hypothesized that when comparing drivers’ decision making in HV-AV interaction and HV-HV interaction, aggressive drivers are more likely to behave aggressively when following an AV and more likely to take advantage of AVs on the road. However, whether an interaction is HV-AV or HV-HV may make no difference in defensive drivers’ decision making. Aggressive drivers were hypothesized to be more likely to make aggressive decisions than defensive drivers in HV-HV interaction and HV-AV interaction. Regarding subjective feelings, it is hypothesized that aggressive drivers have more negative subjective feelings (e.g., anxious, alert, uncomfortable, unsafe) in HV-AV interactions than in HV-HV interactions. However, as with decision making, whether an interaction is HV-AV or HV-HV may make no difference in defensive drivers’ subjective feelings.

## Method

### Participants

Thirty-six participants (18 males and 18 females) took part in this study. All participants were required to have held a driver’s license for at least 2 years. The participants’ ages ranged from 19 to 70 with an average age of 26.52 years (SD = 9.99) and an average annual driving mileage of 8,611 miles (SD = 5156). Of the 36 participants, 12 were classified as aggressive drivers, another 12 as moderate, and still another 12 as defensive according to the Aggressive Driving Scale (ADS; see Section 2.3). Gender was balanced in each driver group. Of the potential participants who completed the ADS in the pre-screening procedure, there were 18 (30.51%) aggressive drivers, 22 (37.29%) moderate drivers, and 19 (32.20%) defensive drivers. To balance the gender differences, in the formal experiment, six male drivers and six female drivers were recruited for each driver group. Participants who completed ADSs were provided with time slots to register for the experiment. The participants were consecutively selected in order of convenience based on their registration sequence. The sampling process concluded when the total number of gender-balanced participants for each driver group was reached. All participants were recruited from the public at large via both Penn State’s StudyFinder website and Facebook groups of prospective participants. This research complied with the American Psychological Association Code of Ethics. Penn State’s Institutional Review Board approved the study. Informed consent was obtained from each participant.

### Apparatus and Stimuli

The driving scenarios in this study were programmed with a driving simulator (STISIM Drive® M300WS-Console) and recorded via Open Broadcaster Software (OBS). The driving simulator was comprised of a Logitech Momo® steering wheel with force feedback (Logitech Inc., Fremont, CA), a throttle pedal, and a brake pedal. The driving scenarios were presented on a 27-inch LCD with 1920×1200-pixel resolution. The vehicles that participants interacted with were programmed to represent the driving styles of an AV and an HV and in different scenarios via STISIM Drive Open Module (OM).

The study was originally designed to have human subjects directly interact with AVs and HVs via the driving simulator. However, given the in-person study challenges and health concerns due to COVID-19, to maintain a consistent human-driving style, a supervised web-based experiment was conducted with a subjective vehicle (SV) that interacted with the AVs or HVs being programmed by STISIM OM. The scenarios were produced by recording the display screens of the driving simulator while it was running each scenario file. The driving videos, pre-video instructions, and post-video questions were then integrated into Qualtrics.

## Materials

*Aggressive Driving Scale (ADS).* This 24-item scale for assessing aggressive driving behaviors was developed by Krahé and Fenske (2002) and validated by Zhang et al. (2016). Participants were asked to report the frequency with which they engaged in particular aggressive behaviors by rating each statement on a 5-point scale ranging from 0 (*never*) to 4 (*very often*). Every participant was pre-screened using the ADS to determine whether their driving style was aggressive, moderate, or defensive. Drivers were classified as aggressive when ADS ≥30 for male drivers and ADS ≥21 for female drivers and defensive when ADS ≤23 for male drivers and ADS ≤13 for female drivers (Krahé, 2005; Krahé & Fenske, 2002).

*Subjective Feelings Towards the Lead Vehicles (AV/HV) Questionnaire*. This survey was developed by the authors (see Supplemental Appendix 1) to measure participants’ comfort, anxiety, alertness, and safety after each car-following scenario on a 9-point scale ranging from 0 (*extremely uncomfortable/calm/unalert/unsafe*) to 8 (*extremely comfortable/anxious/alert/safe*).

*Intention to Behave Aggressively Questionnaire.* This survey included a question that measured participants’ intention to behave aggressively in ways other than following the AV/HV after each car-following scenario (see Supplemental Appendix 2). The question reads: “Do you intend to do anything other than following this lead vehicle (AV/HV)? If yes, can you tell us why you want to do this?”.

*Tendency to Take Advantage of AVs Questionnaire*. This multiple-choice survey was developed by the authors to measure participants’ decision making (whether aggressive or defensive) when interacting with an AV or HV after each more complex scenario (see Supplemental Appendix 3). The choices for each question included one aggressive decision, one defensive decision, and an open option for respondents to indicate any other decisions they would make. An example item is “In this case, if you drive the vehicle, do you intend to wait for the vehicle (AV) from the opposite direction to pass, or turn left without waiting or others?”

### Experiment Design

The experiment adopted a 2 × 3 mixed factorial design with driving style (aggressive vs. moderate vs. defensive) as a between-subject variable and type of road interaction (HV-AV vs. HV-HV) as a within-subject variable. Each participant was required to experience both HV-AV and HV-HV interactions with the order of the interactions being balanced across participants.

There were two blocks in each within-subject experimental condition. The first block (the “car-following” block) was designed to help participants familiarize themselves with the AVs’ driving behavior and measure their subjective feelings and intention to behave aggressively when following an AV or an HV. The second block (the “decision making” block) was designed to explore drivers’ decision making and tendency to take advantage of AVs before they observed the decisions made by the AVs and HVs based on the experience of AVs or HVs in the car-following block.

#### Dependent Variables

There were two sets of dependent variables that were measured in the study: subjective feelings and decision making. Drivers’ *subjective feelings* were measured after each scenario in the car-following block using the *Subjective Feelings Towards the Lead Vehicles Questionnaire*. The questionnaire addressed four constructs. Drivers’ *decision making* was measured with two variables: intention to behave aggressively in car-following scenarios and tendency to take advantage of AVs.

*Intention to behave aggressively* was measured after each scenario in the car-following block (see Table 1). If a participant decided to keep following the lead vehicle, their intention to behave aggressively was coded as “No.” If they indicated they would do anything other than following the lead vehicle, such as overtaking the lead vehicle, changing lanes, or honking, their intention to behave aggressively was coded as “Yes.” However, if they indicated they would not keep following the lead vehicle due to safety concerns, their intention to behave aggressively was coded as “No.” The number of “Yes” responses under the six combined experimental conditions for three driver groups (i.e., aggressive, moderate, and defensive drivers) and across two types of interactions (i.e., HV-AV and HV-HV interactions) was counted as the frequency of a driver’s intention to behave aggressively in car-following scenarios.

**TABLE 1:** Diagrams of Scenarios in Car-Following Block

Scenario	Diagram	AV/HV behavior	Measurements
1. Approaching a intersection at a red light	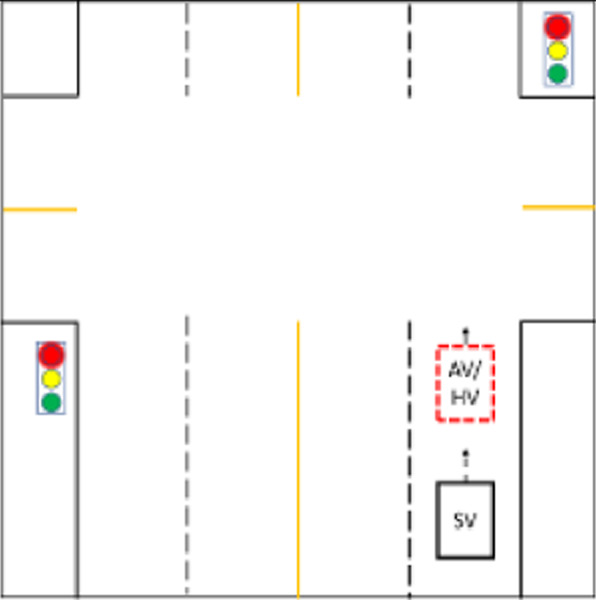	AV waited for 3 seconds after the traffic light changed to green. HV accelerated once the traffic light changed to green.	Driver’s subjective feeling of anxiety, alertness, comfort, and safety The frequency of driver’s intention to behave aggressively
2. AV/HV turning left at the green light and an OV coming from the opposite direction	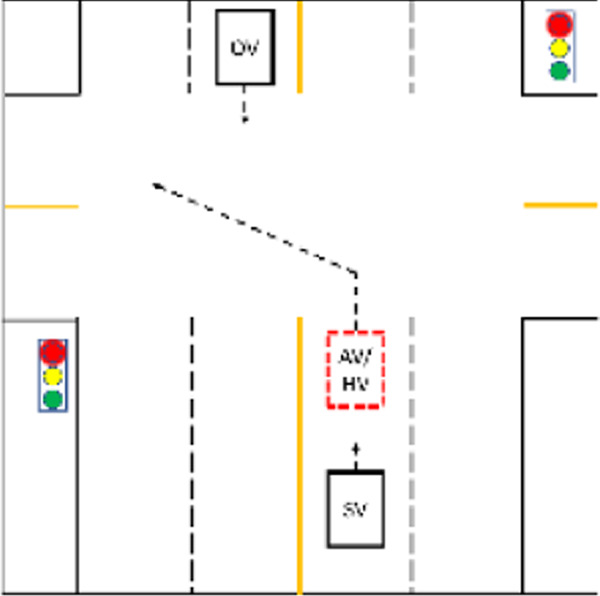	AV stopped and waited until OV passed. HV turned left without waiting.	
3. There was an OV on the right hand arriving later than AV/HV in front of the stop sign.	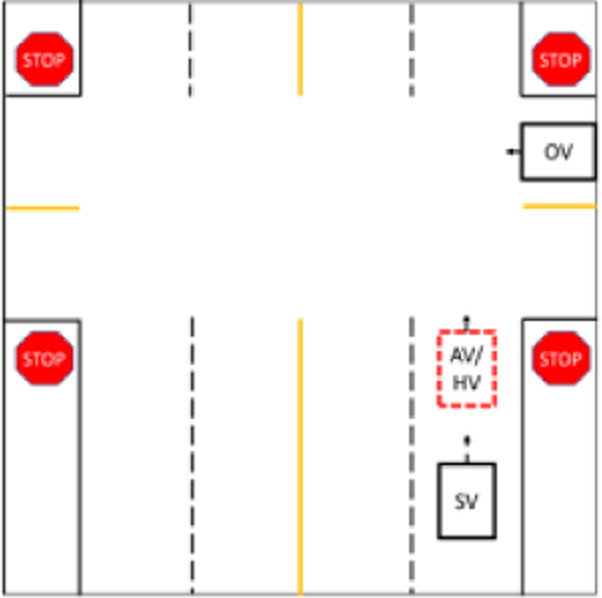	AV waited until OV passed. HV stopped and passed the stop sign before OV moved.	
4. SV followed AV/HV in front of the intersection at the yellow light	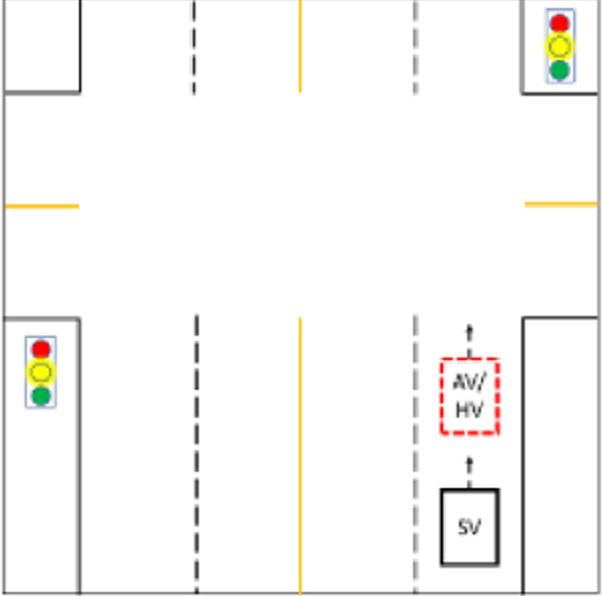	AV stopped and accelerated after the traffic light changed to green. HV passed the intersection without stopping.	
5. There was an OV turning left from the opposite way at the green light	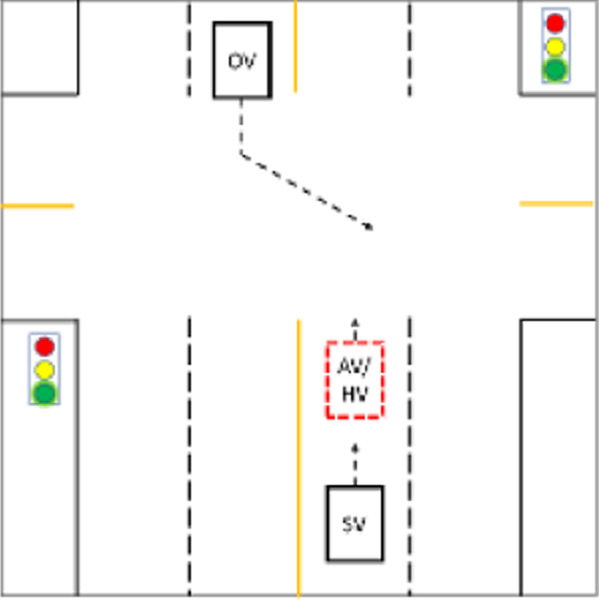	AV stopped and waited until OV passed. HV went straight without stopping.	
6. AV/HV intended to turn right at the red light. There was an OV passing the intersection from left to right	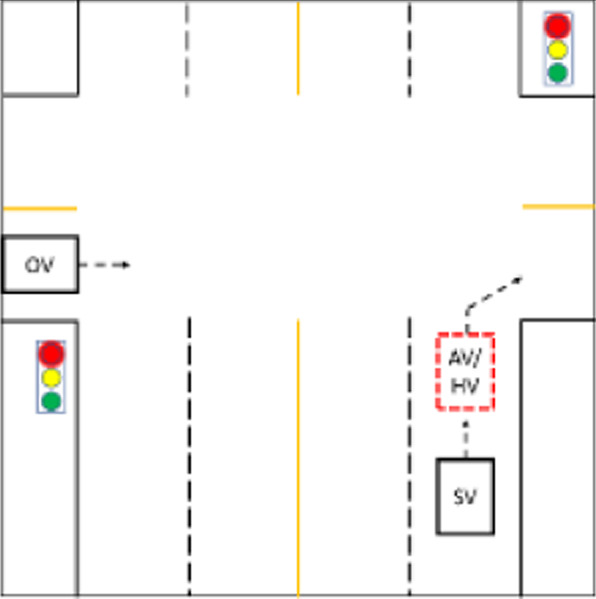	AV stopped and waited until OV passed. HV just stopped for a moment and the turned right before OV arrived.	

*Note:* SV: subjective vehicle; AV/HV: the vehicle directly interacts with the subject vehicle; OV: other vehicles.

*Tendency to take advantage of AVs* was measured after each scenario in the decision making block (see Table 2). Drivers were asked to choose between an aggressive decision and a defensive decision or indicate the alternative decision they would make. The frequency of aggressive decisions was calculated for three driver groups and across two types of interactions. If participants were significantly more likely to make aggressive decisions in HV-AV interactions than in HV-HV interactions, it represented they were more likely to take advantage of AVs than HVs.

**TABLE 2:** Diagrams of Scenarios in Decision Making Block

Scenario	Diagram	AV/HV behavior (same in control level)	Measurements
1. SV intended to turn left and there was an AV/HV coming from the opposite way. (gap = 457 ft)	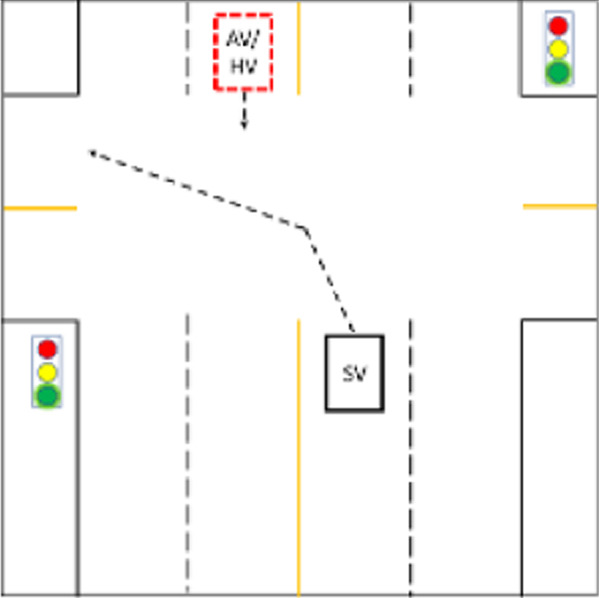	AV/HV drove straightly with the same speed (= 45 mile/h) from the opposite way.	Aggressive decision: Turn left without waiting Defensive decision: Wait until AV/HV passes
2. AV/HV arrived earlier than SV from the right hand in front of the stop sign.	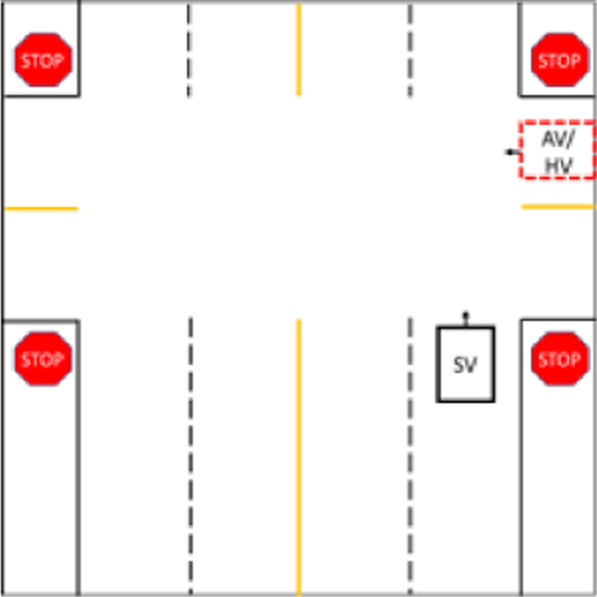	AV/HV arrived at the stop sign earlier than SV with the same speed profile from the left hand.	Aggressive decision: Begin to accelerate before AV/HV starts Defensive decision: Wait until AV/HV passes.
3. SV intended to turn left and there was an AV/HV coming from the opposite way. (gap = 662 ft)	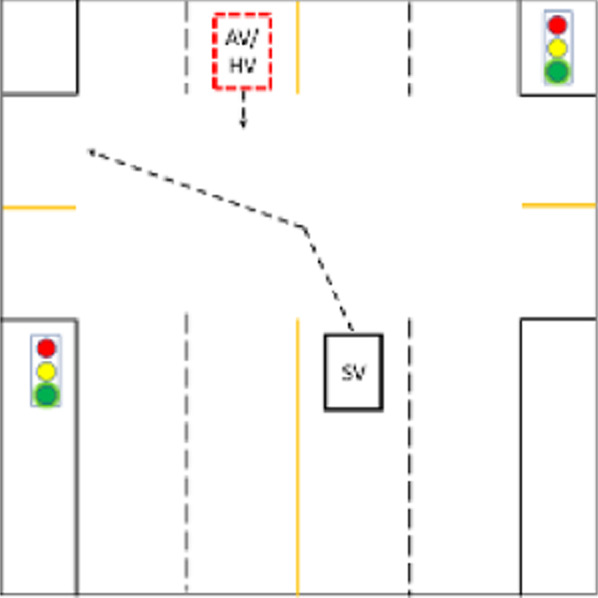	AV/HV drove straightly with the same speed (= 45 mile/h) from the opposite way.	Aggressive decision: Turn left without waiting Defensive decision: Wait until AV/HV passes
4. AV/HV cut in the SV’s lane from the park lane.	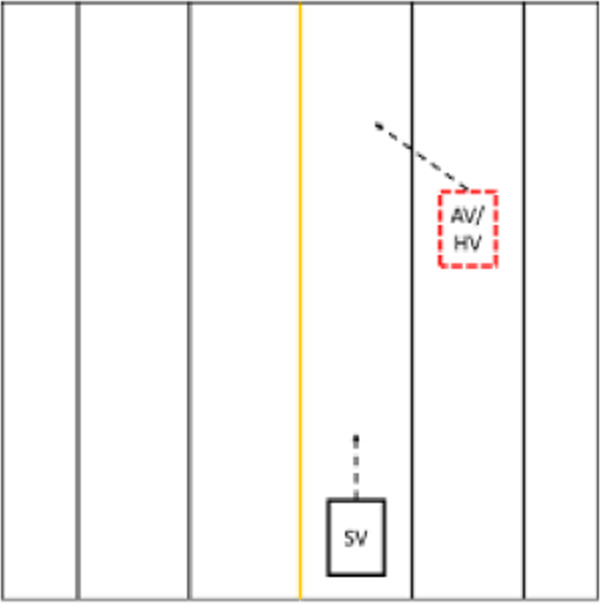	AV/HV turned on the left turning light and moved to the left lane with the same speed for 1 second.	Aggressive decision: Continue driving straight without deceleration Defensive decision: Begin to decelerate to wait for AV cut-in

*Note:* SV: subjective vehicle; AV/HV: the vehicle directly interacts with the subject vehicle; OV: other vehicles.

#### Scenarios

All scenarios were selected and designed based on the reports of 53 AV traffic accidents collected from January 2015 to December 2017 by the California DMV (2018a). All scenarios were selected based on three criteria. First, they address an accident involving HV-AV interaction. Second, they require HV drivers’ decision making at the tactical level. Third, they can be replicated with the STISIM driving simulator.

 Two aspects were considered in designing scenarios for the present study. First, among the reported AV accidents, 64.2% were car-following scenarios and 34.8% were other scenarios, such as sideswiping, colliding broadside, and hitting objects and pedestrians (Favarò et al., 2017; Petrović et al., 2020). Therefore, the present study selected and replicated six of these car-following scenarios in the first block and four other scenarios in the second block. Second, according to the California DMV, 89% of reported AV accidents happened at intersections. Accidents at intersections are associated with drivers’ decision making at the tactical level (Favarò et al., 2017; Petrović et al., 2020). Therefore, nine out of ten designed scenarios involved intersections, whereas one of these scenarios occurred at a straight road segment.

To make the difference between AVs and HVs salient, AVs were shown in a purple color and HVs were designed in a blue color in the driving scenarios. Participants were instructed as to the type of the vehicle (AV vs. HV) they were about to interact with before each scenario. AVs were programmed with a defensive driving style using the driving indicators obtained from a previous study (Ma & Zhang, 2021) similar to those available on the market (Waymo, 2020). Since the present study focused on differences in HV drivers’ decision making in their interaction with AVs and HVs at the tactical level, the control level in Ranney’s three-level hierarchy for HVs and AVs was designed the same. As shown in Table 3, the mean values for each driving indicator and the average decelerations of AVs and HVs were designed the same. The AVs’ deceleration profile was smoother than the HVs’ based on the deceleration profile shared among most participants in a previous study (Ma & Zhang, 2020). The tactical levels of the HVs and AVs were designed to be different. In the car-following block, the SV followed a lead vehicle (i.e., AV or HV) in six scenarios. The AV was more likely than the HV to make defensive decisions (See Table 1). In the decision making block, the SV interacted with an AV or an HV on the road in four different scenarios. Each video was paused right before the AV or HV showed their decisions when they interacted with the SV (See Table 2).

**TABLE 3:** Values of Driving Indicators for AVs and HVs

	AVs	HVs
Average speed (mph)	45	45
Acceleration (ft/s^2^)	5.468	5.468
Average deceleration (ft/s^2^)	−3.712	−3.712
Deceleration profile	3.712 ft/s^2^ for 17.78 s	6.26 ft/s^2^ for 2.94 s, 5.48 ft/s^2^ for 2.21 s, 3.91 ft/s^2^ for 2.27 s, 2.35 ft/s^2^ for 2 s, keep 22.06 ft/s for 3.5 s, 4.54 ft/s^2^ for 4.86 s

**Figure 1.**
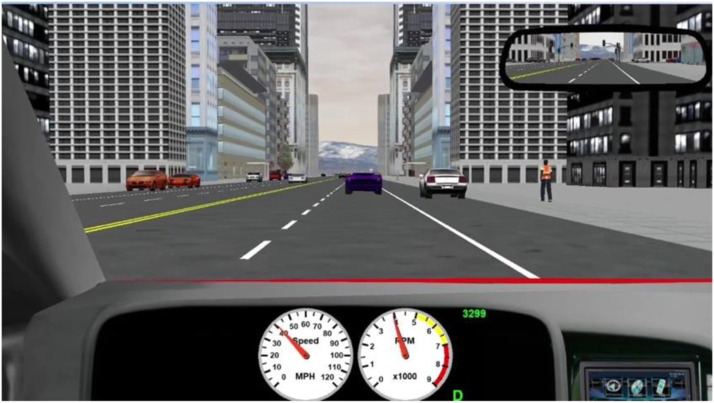
Screenshot of urban scenario.

The sequence of scenarios was balanced with a Latin square design in each block to reduce the sequential effect of scenarios. As shown in Figure 1, scenarios were presented from the perspective of the HV drivers. An urban environment was simulated with two lanes in each direction for a given roadway, moderate traffic density (13 vehicles per mile per lane), dense buildings, and pedestrians on the sidewalk. The posted speed limit was 45 miles per hour (mph).

### Procedure

The web-based experiment was conducted and overseen using Zoom, a conferencing software. Before the formal experiment began, the experiment instructions, recorded driving scenarios, and questionnaires were integrated in Qualtrics. Potential participants’ driver’s licenses were checked via the web camera to confirm the individuals’ eligibility to participate in the experiment. The participants were asked to indicate their willingness to participate in the experiment and consent to video recording. They were not required to turn on their web cameras during the experiment, but they were required to share their screens via Zoom when filling out the questionnaires. The participants received instruction on the capabilities of Level 5 AVs: “For the purposes of this study, the term ‘automated vehicle’ refers to a car that can drive itself. The operation of the vehicle would be the driver entering a destination, and the vehicle would operate itself to the final destination with no input from the driver. The driver does, however, have the ability to take over the operation of the vehicle. Thus, the driver can operate the vehicle, but does not have to drive under any situation of the vehicle’s operation.”

**Figure 2.**
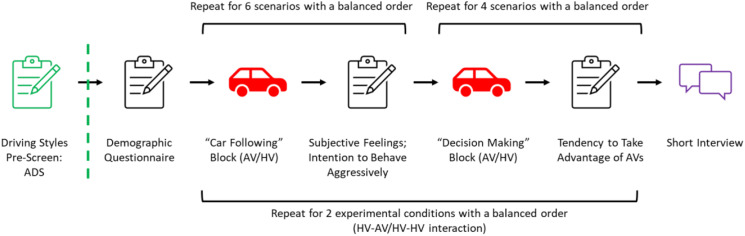
Experimental procedure.

As shown in Figure 2, the participants were asked to fill out various demographic questionnaires. In the formal experiment, the participants were instructed to imagine manually driving the SV in a city, to interact with AVs and HVs by watching the recorded driving scenarios, and to evaluate their experience with HV-AV and HV-HV interactions. The order of the HV-AV and HV-HV interaction blocks was balanced and predefined based on the participants’ registration sequence. In each experimental condition (HV-AV or HV-HV), there were two blocks. In the car-following block, the participants experienced six scenarios in which they followed an AV or an HV depending on the experimental condition. After experiencing each scenario, the participants were required to fill out the *Subjective Feelings Towards the Lead Vehicles (AV/HV) Questionnaire* measuring their subjective feelings and the *Intention to Behave Aggressively Questionnaire* measuring their intention to behave aggressively. In the decision making block, the participants interacted with an AV or an HV in four scenarios depending on the experimental condition. After each scenario, they were required to fill out the *Decision Making Questionnaire*. Finally, they took part in a short interview designed to investigate any differences they found when interacting with the two different types of vehicles. Between blocks, participants could take a short break as needed. The total experiment time for each participant was 45–60 min.

### Data Analysis

Due to the normality and homogeneity of variance assumptions of ANOVAs being violated, mixed ANOVAs with a bootstrapping approach (B = 1000) were conducted to analyze the effect of driving style (i.e., aggressive, moderate, or defensive) and type of interaction (HV-AV vs. HV-HV) on drivers’ subjective feelings (Field, 2013; Xu et al., 2013).

*Bootstrapping* is a statistical method that uses random sampling with replacement. It is an alternative to traditional hypothesis tests that does not require these assumptions (Šaltytė Benth et al., 2008). The achieved significance level (i.e., *p*-value) was estimated and reported by calculating the proportion of the value of test statistics from bootstrapping that was greater than or equal to the original value of test statistics (Walters & Campbell, 2004). A bootstrap procedure was conducted for the multiple comparisons based on Dunnett (1955) procedure (MacKinnon, 2009; Westfall, 2011) to analyze drivers’ subjective feelings between each pair of driving styles. The procedure has shown below:

1. Suppose we have *m* multiple comparisons with *m* test statistics, $\tau_{i}$ , i=1,…,m . $\tau_{max}\equiv max\left(\tau_{i}\right)$ .
2. Calculate all *m* test statistics for the actual sample and each bootstrap sample (B = 1000) to get $\tau_{max}^{*}$ .
3. Compute 1−α quantile of $\tau_{max}^{*}$ and compare it with each test statistics for the actual sample ${\hat{\tau }}_{i}$ .

Based on the steps above, *p* values can be obtained to decide if researchers could reject the null hypothesis with multiple comparisons to avoid alpha inflation.

Mixed-effects logistic regression models with random effects were constructed to analyze the frequency of drivers’ intentions to behave aggressively in the car-following block and the frequency of drivers’ aggressive decision making in the decision making block. If participants were significantly more likely to make aggressive decisions in HV-AV interactions than in HV-HV interactions, it represented they were more likely to take advantage of AVs than HVs. Odds ratio (OR) was reported as the standardized effect size statistics for logistic regression models because the independent variables in this study were categorical variables (Schumacker, 2005). Due to drivers’ driving style having three categories, two ORs were reported as the effect size statistics for the main effect of this independent variable, including OR_AD_ (i.e., odds ratio between aggressive and defensive drivers) and OR_MD_ (i.e., odds ratio between moderate and defensive drivers). One OR was reported as the effect size statistics for the main effect of the other independent variable, the type of interaction.

Due to the lack of an operationalized structure for the interview question, all participants’ responses were subjected to inductive content analysis (Lauri & Kyngas, 2005) via NVivo 1.4.1. Every sentence in the participants’ responses was analyzed using an open coding method in which the headings were recorded (Elo & Kyngäs, 2008). These headings were subsequently compiled and used to generate the categories of perceived differences between AVs and HVs (Burnard, 1991). The frequency of each generated category was reported.

## Results

The demographics and driving history information of each driver group are shown in Table 4. Kruskal–Wallis H tests were conducted to analyze the demographic differences among the three groups. The results showed that there was a significant difference in driver age (*H*(2) = 7.34, *p* = .03) that might have been caused by two outliers (i.e., two participants who were much older than the others) in the defensive group. After excluding these outliers, no significant difference in driver age (*H*(2) = 4.62, *p* = .99) was found. There was no significant difference in annual driving mileage (*H*(2) = .46, *p* = .80) or length of time holding a driver’s license (*H*(2) = .37, *p* = .83), either. A logistic regression model was constructed to analyze the differences in moving violations among the three groups. The results showed that there was a significant difference in moving violations (*χ*^2^(2) = 10.21, *p* = .006).

**TABLE 4:** Demographic Information for Each Driver Group (Mean ± SE)

	Aggressive drivers	Moderate drivers	Defensive drivers
Age (years)	23.50 ± 1.20	25.75 ± 1.78	32.33 ± 4.15
Annual driving mileage (miles)	8541 ± 1357	8333 ± 1749	8958 ± 1458
Driver license age (years)	4.92 ± .76	6.29 ± 1.68	12.33 ± 4.73
ADS score	34.67 ± 2.87	21.00 ± 1.65	11.42 ± 1.93
Type of car	41.67% SUV, 50% sedan or Wagon, 8.33% truck or pick-up	25% SUV, 41.67% sedan or Wagon, 25% 2-door coupe or hatchback, 16.67% van or minivan	33.33% SUV, 66.67% sedan or Wagon, 8.33% 2-door coupe or hatchback
Moving violations	83.33%	33.33%	25%

### Subjective Feelings

#### Anxiety

**Figure 3.**
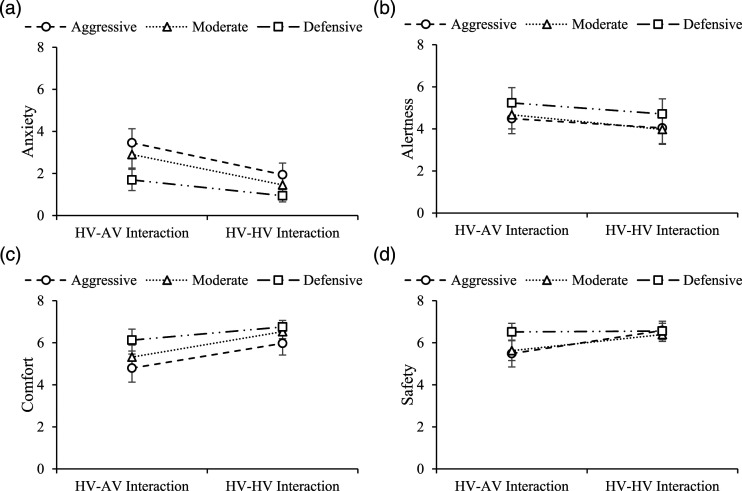
Effects of driving style and type of interaction on drivers’ subjective feelings (error bars: ± 1 SE).

As shown in Figure 3(a), a significant main effect of type of interaction on driver anxiety (*p* = .01) was found. For pairwise comparisons, as shown in Table 5, aggressive (*p* = .004) and moderate (*p* = .006) drivers felt significantly more anxious when following an AV than an HV. A significant main effect of driving style on anxiety when following a lead vehicle was also found (*p* = .02). The analysis of pairwise comparisons suggested that, as shown in Table 6, aggressive drivers felt significantly more anxious in both HV-AV (*p* = .01) and HV-HV (*p* = .047) interactions than defensive drivers. No significant interaction effect on driver anxiety was found (*p* = .43).

**TABLE 5:** Pairwise Comparison of Each Subjective Feeling for Drivers With Same Driving Style

Subjective feeling	Drivers’ driving style	Pairwise comparison
Anxiety	Aggressive	AV vs. HV: *p* = .004**
	Moderate	AV vs. HV: *p* = .006**
	Defensive	AV vs. HV: *p* = .12
Alertness	Aggressive	AV vs. HV: *p* = .42
	Moderate	AV vs. HV: *p* = .06
	Defensive	AV vs. HV: *p* = .40
Comfort	Aggressive	AV vs. HV: *p* = .009**
	Moderate	AV vs. HV: *p* = .03*
	Defensive	AV vs. HV: *p* = .25
Safety	Aggressive	AV vs. HV: *p* = .04*
	Moderate	AV vs. HV: *p* = .06
	Defensive	AV vs. HV: *p* = .87

*Note:* * *p* < .05, ** *p* < .01, *** *p* < .001.

**TABLE 6:** Pairwise Comparison of Each Subjective Feeling for Drivers Having Same Type of Interaction

Subjective feeling	Type of interaction	Pairwise comparison
Anxiety		Aggressive vs. Moderate: *p* = .43
	HV-AV	Aggressive vs. Defensive: *p* = .01**
		Moderate vs. Defensive: *p* = .14
		Aggressive vs. Moderate: *p* = .31
	HV-HV	Aggressive vs. Defensive: *p* = .047*
		Moderate vs. Defensive: *p* = .12
Alertness		Aggressive vs. Moderate: *p* = .84
	HV-AV	Aggressive vs. Defensive: *p* = .42
		Moderate vs. Defensive: *p* = .57
		Aggressive vs. Moderate: *p* = .94
	HV-HV	Aggressive vs. Defensive: *p* = .53
		Moderate vs. Defensive: *p* = .45
Comfort		Aggressive vs. Moderate: *p* = .43
	HV-AV	Aggressive vs. Defensive: *p* = .07
		Moderate vs. Defensive: *p* = .24
		Aggressive vs. Moderate: *p* = .23
	HV-HV	Aggressive vs. Defensive: *p* = .10
		Moderate vs. Defensive: *p* = .49
Safety		Aggressive vs. Moderate: *p* = .79
	HV-AV	Aggressive vs. Defensive: *p* = .047*
		Moderate vs. Defensive: *p* = .048*
		Aggressive vs. Moderate: *p* = .60
	HV-HV	Aggressive vs. Defensive: *p* = .94
		Moderate vs. Defensive: *p* = .64

*Note:* * *p* < .05, ** *p* < .01, *** *p* < .001.

#### Alertness

As shown in Figure 3(b), no significant main effects of driving style (*p* = .68), type of interaction (*p* = .06), or interaction effect (*p* = .94) on driver alertness were found.

#### Comfort

As shown in Figure 3(c), a significant main effect of type of interaction on driver comfort when following a lead vehicle was found (*p* = .005). The analysis of pairwise comparisons suggests that aggressive (*p* = .009) and moderate (*p* = .03) drivers felt significantly more comfortable when following an HV than an AV. No significant main effects of driving style (*p* = .07) or interaction effect (*p* = .61) on driver comfort were found.

#### Safety

As shown in Figure 3(d), a significant main effect of type of interaction on driver safety when following a lead vehicle was found (*p* = .01). The analysis of pairwise comparisons suggests that aggressive drivers felt significantly safer when following an HV than an AV (*p* = .04). Defensive drivers felt significantly safer in HV-AV interaction than either aggressive drivers (*p* = .047) or moderate drivers (*p* = .048). No significant main effects of driving style (*p* = .23) or interaction effect (*p* = .17) on driver safety were found.

### Intention to Behave Aggressively

**Figure 4.**
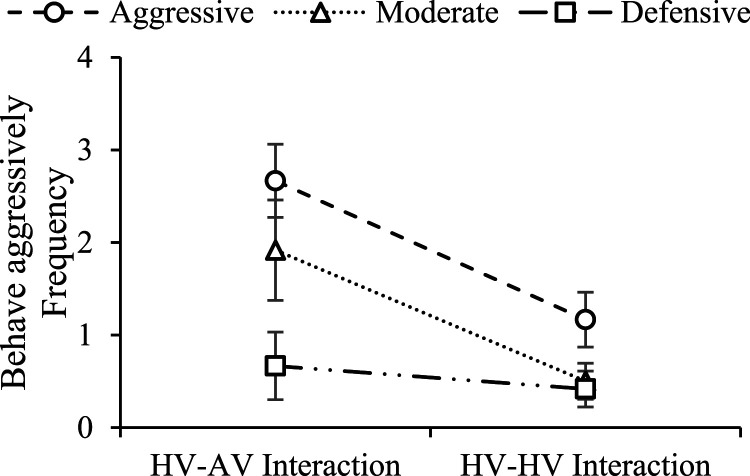
Effects of driving style and type of interaction on drivers’ intention to behave aggressively (error bars: ± 1 SE).

As shown in Figure 4, the results reveal a significant main effect of driving style on frequency of intention to behave aggressively when following a lead vehicle (*χ*^2^(2) = 10.43, *p* = .005, OR_AD_ = 9.22, OR_MD_ = 3.07). For pairwise comparisons, aggressive drivers were significantly more likely to behave aggressively than defensive drivers in HV-AV (*χ*^2^(1) = 12.02, *p* < .001, OR = 14.95) and HV-HV interactions (*χ*^2^(1) = 4.17, *p* = .04, OR = 5.69). Moderate drivers were significantly more likely to behave aggressively than defensive drivers in HV-AV interaction (*χ*^2^(1) = 6.41, *p* = .01, OR = 7.05). A significant main effect of type of interaction on frequency of intention to behave aggressively was also found (*χ*^2^(1) = 20.02, *p* < .001, OR = 4.64). For pairwise comparisons, aggressive (*χ*^2^(1) = 12.83, *p* < .001, OR = 5.08) and moderate (*χ*^2^(1) = 15.39, *p* < .001, OR = 10.19) drivers were significantly more likely to behave aggressively in HV-AV interaction than HV-HV interaction, whereas no such difference was found for defensive drivers (*χ*^2^(1) = .95, *p* = .32). No significant interaction effect was found (*χ*^2^(2) = 3.50, *p* = .17).

### Tendency to Take Advantage of AVs

**Figure 5.**
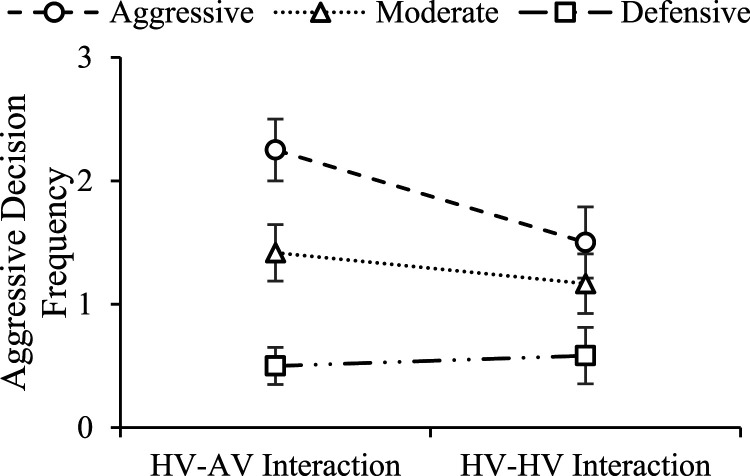
Effects of driving style and type of interaction on drivers’ aggressive decision making (error bars: ± 1 SE).

As shown in Figure 5, the results indicate a significant main effect of driving style on frequency of aggressive decision making given the opportunity to take advantage of a certain vehicle (*χ*^2^(2) = 21.08, *p* < .001, OR_AD_ = 9.07, OR_MD_ = 4.37). For pairwise comparisons, aggressive drivers were significantly more likely to make aggressive decisions than defensive drivers in HV-AV (*χ*^2^(1) = 19.36, *p* < .001, OR = 16.41) and HV-HV interactions (*χ*^2^(1) = 7.12, *p* = .008, OR = 5.01). Moderate drivers were significantly more likely to make aggressive decisions than defensive drivers in HV-AV interaction (*χ*^2^(1) = 8.51, *p* = .004, OR = 6.22). No significant main effects of type of interaction (*χ*^2^(1) = 1.74, *p* = .19) or interaction effect (*χ*^2^(2) = 2.17 *p* =.34) on the frequency of aggressive decision making were found.

The tendency to take advantage of AVs was analyzed by comparing the frequency of aggressive decision making in HV-HV interaction to that in HV-AV interaction. The results showed aggressive drivers were significantly more likely to make aggressive decisions in HV-AV interaction than in HV-HV interaction (*χ*^2^(1) = 4.24, *p* = .04, OR = 2.64), indicating that aggressive drivers are more likely to take advantage of AVs than HVs. No such effect was found for moderate drivers or defensive drivers.

### Perceived Differences Between AVs and HVs

**Figure 6.**
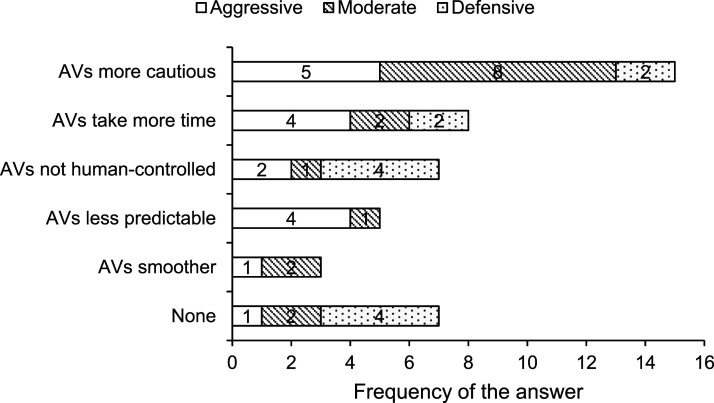
Frequency with which each difference between AVs and HVs was categorized.

In all, six categories regarding the participants’ different perceptions of AVs and HVs were identified, and the frequency for each category was calculated. As shown in Figure 6, there were five major differences between AVs and HVs reported by the participants, including “AVs are more cautious,” “AVs take more time,” “AVs are not controlled by humans,” “AVs are less predictable,” and “AVs drive more smoothly.” Seven (19.44%) participants did not identify any differences between AVs and HVs.

As shown in Table 7, aggressive drivers considered AVs were more cautious and required more time to make decisions. This may be due to the fact that aggressive drivers drive in a different style than defensive AVs at the tactical level. Some aggressive drivers (*n* = 4) thought AVs drove more unpredictably because AVs’ defensive driving behavior was significantly different from their own. Most moderate drivers perceived the difference between HVs and AVs as AVs being more cautious than HVs, which might indicate the moderate drivers focused more on other road users’ specific driving behaviors than other driver groups. Defensive drivers cared more than the other two groups of drivers that the agent of AVs was not human. A third of the defensive drivers did not identify any differences between AVs and HVs, which might suggest that they focused on their own driving behaviors and were courteous to other road users (Sagberg et al., 2015). Moreover, some defensive drivers (*n* = 4) felt more cautious in HV-AV interaction than they did in HV-HV interaction because they were not able to communicate with AVs in some situations.

**TABLE 7:** Frequency and Examples of Comments Made by Drivers in Each Group

Driver’s driving style	Main comment	Frequency	Example
Aggressive	AVs are more cautious	5	“AV takes every move cautiously which means safe and slow.”
	AVs take more time	4	
	AVs are less predictable	4	“I think during the HV, it was much more predictable what the car was going to do because it followed the same driving guidelines that I as a human follow. Therefore, I felt less attentive and nervous around the HV car. However, the AV is more unpredictable (like stopping at a green light, not turning on red) that I felt more alert.”
Moderate	AVs are more cautious	8	“It was clear that the AV drove more cautiously. The HV sped up through a yellow light and made a right turn on red while the AV did not do those things because it was clearly programmed to be very careful.”
Defensive	No difference between AVs and HVs	4	
	AVs are not controlled by humans	4	“I can’t give a signal to AV. On the stop sign, I could give a signal to people if I want to go first or let him/her go.”

## Discussion

### Main Findings

This study investigated the impact of driving style (i.e., aggressive, moderate, and defensive) and type of interaction (HV-AV vs. HV-HV) on HV drivers’ subjective feelings, intention to behave aggressively, and tendency to take advantage of AVs. The results indicate that aggressive drivers felt significantly more anxious, uncomfortable, and unsafe and were more likely to behave aggressively in HV-AV interaction than in HV-HV interaction. Moderate drivers felt significantly more anxious and uncomfortable and were also more likely to behave aggressively in HV-AV interaction than in HV-HV interaction. No such differences were found for the defensive drivers in this study, indicating they were not significantly influenced by the type of interacting vehicle. Moreover, aggressive drivers were more likely to take advantage of interacting vehicles in HV-AV interaction than in HV-HV interaction. There was no difference in moderate and defensive drivers’ decision making between HV-AV interaction and HV-HV interaction.

In the present study, the AVs were designed to make more defensive decisions than the average HV driver. The differences between the AVs’ defensive driving style and aggressive and moderate drivers’ driving styles at the tactical level (e.g., AVs slowing down more frequently than expected) led to more anxious feelings and more frequent aggressive behaviors, which might cause an increase in collisions in mixed traffic. This finding is consistent with that of Ye and Yamamoto (2019). In their simulations, Ye and Yamamoto found that differences in AVs’ and HVs’ behaviors at the control level negatively influence traffic safety. Compared with Ye and Yamamoto (2019)’s study that simulated HV and AV control performance in simple car-following scenarios, this study focused on drivers with different driving styles and decision making tendencies regarding HVs and AVs and investigated HV-AV interaction in various scenarios including intersections. In a conventional transportation system, aggressive drivers attract attention due to their risky behavior. However, this study indicates that moderate drivers may behave more aggressively in mixed traffic, which suggests a need for improved driver safety in mixed traffic. This finding expands on those of Liu et al. (2020). In their survey study, Liu et al. found drivers were more likely to behave aggressively in HV-AV interaction than in HV-HV interaction; however, they did not consider drivers’ driving styles. The findings of this study indicate that only aggressive and moderate drivers showed changes in their intentions to behave more aggressively in HV-AV interaction.

In terms of the tendency to take advantage of AVs, the present study found that aggressive drivers were more likely to take advantage of AVs than HVs. This finding corroborates the speculation in Tennant et al.’s (2016) survey study. Tennant et al. found that drivers who behaved more aggressively toward other road users were more optimistic about AVs. The researchers explained the results by speculating that aggressive drivers may perceive AVs to be easier to take advantage of than human drivers. This finding also helps explain the results of Trende et al. (2019)’s simulator study. In Trende et al. (2019)’s study and the present study, AVs and HVs behaved similarly at the control level and did not show their decision making at the tactical level before participants made decisions. Trende et al. (2019) found that no significant evidence indicating drivers were more likely to take advantage of AVs without time pressure, and the results from the current study indicate only aggressive drivers are more likely to take advantage of AVs than HVs. The proportion of aggressive drivers might be less than one third of the normal population (AAA, 2019), which might be why Trende et al. found no significant effect without time pressure. Moreover, the results of the car-following block indicate both the aggressive and moderate drivers intended to behave more aggressively in HV-AV interaction than in HV-HV interaction after observing lead vehicles’ decision making and driving behavior. The results of the decision making block indicate that the aggressive drivers tended to make more aggressive decisions based on their experience with AVs and HVs in the previous block rather than other vehicles’ specific behaviors. One possible explanation is that aggressive drivers focus more on accomplishing their travel goals with the intention to reach the destinations faster (Sagberg et al., 2015), so they make aggressive decisions with less information and prefer to take advantage of other road users.

### Practical Implications

The present study reveals that drivers’ subjective feelings, intention to behave aggressively, and tendency to take advantage of AVs may be influenced significantly by the drivers’ own driving styles. Today the development of AVs focuses on road object detection and behavior prediction based on the classification of pedestrians, cyclists, and motorcyclists (Waymo, 2020). The present study highlights the importance of AVs understanding and proactively predicting drivers’ decisions based on the drivers’ driving styles to avoid traffic conflicts between HVs and AVs. Another implication of this study is the need to improve AVs’ decision making algorithm to be more human-like and thereby mitigate the aggressive behaviors of HVs in mixed traffic. Waytz et al. (2014) argued for the development of human-like automated systems that could improve drivers’ trust in AVs. The present study also suggests the necessity of developing proper training and education programs to enhance drivers’ awareness of potential changes in their own behavior in HV-AV interaction that may lead to hazards.

### Limitations and Future Research

Although this study was carefully conducted, there are several limitations. Since the present study was a web-based experiment, drivers’ intentions in decision making were collected via post-scenario questions, but their actual driving performance was not measured. Behavioral experiments need to be conducted in order to better understand how drivers actually make decisions and behave in HV-AV interaction. Second, the AVs in the present study adopted a defensive driving style; however, AVs can adopt different driving styles that may influence driver behavior in HV-AV interaction on the road. This study could be repeated using different AV algorithms and the results could be compared to determine the optimal AV driving style for HV-AV interactions. Thirdly, participants’ understanding of AVs was not assessed directly after they interacted with AVs. Only the perceived differences between AVs and HVs were measured during the experiment. Future work is recommended to measure drivers’ understanding of the capacities and limitations of the imagined AV. In addition, participants were informed about the types of the vehicles that they interacted with in this study. Future research might investigate drivers’ subjective feelings, decision making, and driving behavior without informing them about the type of vehicles with which they are interacting. In this way, researchers could determine whether drivers can perceive behavioral differences between HVs and AVs.

## Conclusion

In summary, the present study reveals that driving style (i.e., aggressive, moderate, or defensive) and type of interaction (HV-AV vs. HV-HV interaction) influenced the participating drivers’ subjective feelings and decision making. This study provides insight into how human drivers perceive and interact with AVs and HVs on the road and how human drivers take advantage of AVs. The findings of the study provide a foundation for developing guidelines for mixed transportation systems to improve drivers’ safety and user experience. In particular, AVs need to observe the decision making and driving behavior of other vehicles at the tactical level and adjust their own behavior accordingly. This would reduce traffic conflicts in HV-AV interaction and improve the safety of the mixed transportation system. Moreover, driver training and education should be made available to drivers so that they understand the potential safety issues in HV-AV interaction.

## Supplemental Material

**Supplemental Material -** Driver-Automated Vehicle Interaction in Mixed Traffic: Types of Interaction and Drivers’ Driving Styles Click here for additional data file. Supplemental Material for Driver-Automated Vehicle Interaction in Mixed Traffic: Types of Interaction and Drivers’ Driving Styles by Zheng Ma and Yiqi Zhang in Human Factors
