# Supplementary material for: Driver-Automated Vehicle Interaction in Mixed Traffic: Types of Interaction and Drivers’ Driving Styles
Source: Hum Factors. 2022 Apr 25;66(2):544–61. doi: 10.1177/00187208221088358 (PMC10757400; doi:10.1177/00187208221088358)
Supplement: Supplemental Material - Driver-Automated Vehicle Interaction in Mixed Traffic: Types of Interaction and Drivers’ Driving Styles [file sj-pdf-1-hfs-10.1177_00187208221088358.pdf]

Appendix 1.

Subjective Feelings towards the Lead Vehicles (AV/HV)

Now we will begin the formal session of the experiment. Please imagine that you are driving a vehicle manually in a city and following a purple autonomous vehicle (AV)/ a blue human-driven vehicle (HV). The speed limit is 45 mph. After watching each video, please indicate how you feel towards the lead vehicle (AV/HV) by answering the following questions.

Instruction: Please **use the full screen** to watch the video and pick the best answer that indicates **how you felt towards the lead vehicle (AV/HV) during this specific event**.

1. How comfortable did you feel when following this lead vehicle (AV/HV)?

Extremely Uncomfortable								Extremely Comfortable
0	1	2	3	4	5	6	7	8

2. How much anxiety did you feel when following this lead vehicle (AV/HV)?

Extremely Not Anxious								Extremely Anxious
0	1	2	3	4	5	6	7	8

3. How much alertness did you feel when following this lead vehicle (AV/HV)?

Extremely Not Alert								Extremely Not Alert
0	1	2	3	4	5	6	7	8

4. How much safety did you feel when following this lead vehicle (AV/HV)?

Extremely Unsafe								Extremely Safe
0	1	2	3	4	5	6	7	8

Appendix 2

Intention to behave aggressively

Do you intend to do anything other than following this lead vehicle (AV/HV)?

If yes, can you tell us why you want to do this?

Appendix 3

Tendency to take advantage of AVs

Please imagine that you are driving a vehicle manually in a city. In this session, you will interact with the same purple autonomous vehicle (AV)/ a blue human-driven vehicle (HV) via different scenarios. The speed limit is 45 mph. After watching each video, please indicate how you would make decisions when interacting with the purple AV/ blue HV by answering the following questions based on your previous experience.

Instruction: Please **use the full screen** to watch the video and pick the best answer that indicates **how you will behave when interacting with the AV/HV during this specific event**.

Scenario 1: In this case, if you drive the vehicle, do you intend to wait for the vehicle (AV/HV) from the opposite direction to pass, or turn left without waiting?

- Wait until AV/HV passes
- Turn left without waiting
- Other:
- If you would like to explain your reasoning, please do so:

Scenario 2: In this case, if you drive the vehicle, do you intend to wait for the vehicle (AV/HV) from the crossing line pass, or would you accelerate before it starts?

- Wait until AV/HV passes
- Begin to accelerate before AV/HV starts
- Other:
- If you would like to explain your reasoning, please do so:

Scenario 3: In this case, if you drive the vehicle, do you intend to wait for the vehicle (AV/HV) from the opposite direction to pass, or turn left without waiting?

- Wait until AV/HV passes
- Turn left without waiting
- Other:
- If you would like to explain your reasoning, please do so:

Scenario 4: In this case, if you drive the vehicle, do you intend to decelerate to wait for the vehicle (AV/HV) from the parking lane to cut in, or continue driving straight without deceleration?

- Begin to decelerate to wait for AV/HV to cut-in
- Continue driving straight without deceleration
- Other:
- If you would like to explain your reasoning, please do so:

Appendix 4

Interview Question

Do you think there are any differences between AV and HV during your driving task? If yes, can you tell us?
